# Supplementary material for: Assessing affective valence and activation in resistance training with the feeling scale and the felt arousal scale: A systematic review
Source: PLoS One. 2023 Nov 16;18(11):e0294529. doi: 10.1371/journal.pone.0294529 (PMC10653427; doi:10.1371/journal.pone.0294529)
Supplement: S1 File — (DOCX) [file pone.0294529.s002.docx]

| Description | Search terms |
| --- | --- |
| Physical: | "physical examination"[MeSH Terms] OR ("physical"[All Fields] AND "examination"[All Fields]) OR "physical examination"[All Fields] OR "physical"[All Fields] OR "physically"[All Fields] OR "physicals"[All Fields] |
| Exercise: | "exercise"[MeSH Terms] OR "exercise"[All Fields] OR "exercises"[All Fields] OR "exercise therapy"[MeSH Terms] OR ("exercise"[All Fields] AND "therapy"[All Fields]) OR "exercise therapy"[All Fields] OR "exercise's"[All Fields] OR "exercised"[All Fields] OR "exerciser"[All Fields] OR "exercisers"[All Fields] OR "exercising"[All Fields] |
| Activity: | "activable"[All Fields] OR "activate"[All Fields] OR "activated"[All Fields] OR "activates"[All Fields] OR "activating"[All Fields] OR "activation"[All Fields] OR "activations"[All Fields] OR "activator"[All Fields] OR "activator's"[All Fields] OR "activators"[All Fields] OR "active"[All Fields] OR "actived"[All Fields] OR "actively"[All Fields] OR "actives"[All Fields] OR "activities"[All Fields] OR "activity's"[All Fields] OR "activitys"[All Fields] OR "motor activity"[MeSH Terms] OR ("motor"[All Fields] AND "activity"[All Fields]) OR "motor activity"[All Fields] OR "activity"[All Fields] |
| Feeling: | "emotions"[MeSH Terms] OR "emotions"[All Fields] OR "feeling"[All Fields] OR "feelings"[All Fields] OR "feels"[All Fields] |
| Scale: | "scale's"[All Fields] OR "scaled"[All Fields] OR "scaling"[All Fields] OR "scalings"[All Fields] OR "weights and measures"[MeSH Terms] OR ("weights"[All Fields] AND "measures"[All Fields]) OR "weights and measures"[All Fields] OR "scale"[All Fields] OR "scales"[All Fields] |
| Arousal: | "arousability"[All Fields] OR "arousable"[All Fields] OR "arousal"[MeSH Terms] OR "arousal"[All Fields] OR "arousals"[All Fields] OR "arousal's"[All Fields] OR "arouse"[All Fields] OR "arouses"[All Fields] OR "wakefulness"[MeSH Terms] OR "wakefulness"[All Fields] OR "aroused"[All Fields] OR "arousing"[All Fields] |
| Resistance: | "resist"[All Fields] OR "resistance"[All Fields] OR "resistances"[All Fields] OR "resistant"[All Fields] OR "resistants"[All Fields] OR "resisted"[All Fields] OR "resistence"[All Fields] OR "resistences"[All Fields] OR "resistent"[All Fields] OR "resistibility"[All Fields] OR "resisting"[All Fields] OR "resistive"[All Fields] OR "resistively"[All Fields] OR "resistivities"[All Fields] OR "resistivity"[All Fields] OR "resists"[All Fields] |
| Strength: | "strength"[All Fields] OR "strengths"[All Fields] |
| Training: | "education"[Subheading] OR "education"[All Fields] OR "training"[All Fields] OR "education"[MeSH Terms] OR "train"[All Fields] OR "train's"[All Fields] OR "trained"[All Fields] OR "training's"[All Fields] OR "trainings"[All Fields] OR "trains"[All Fields] |
