# Supplementary figures and images for: Assessing affective valence and activation in resistance training with the feeling scale and the felt arousal scale: A systematic review
Source: PLoS One. 2023 Nov 16;18(11):e0294529. doi: 10.1371/journal.pone.0294529 (PMC10653427; doi:10.1371/journal.pone.0294529)

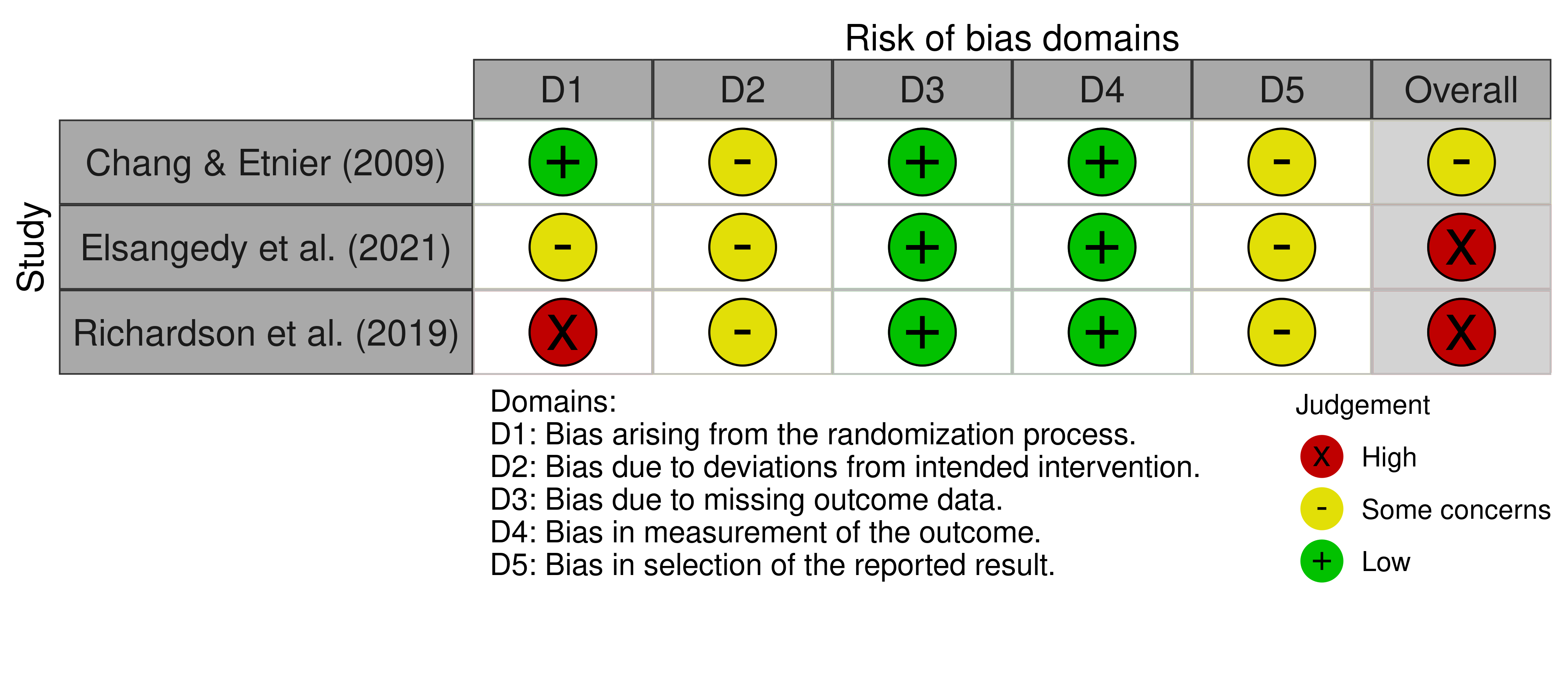

Supplement: S2 File — (TIF) [file pone.0294529.s003.tif]

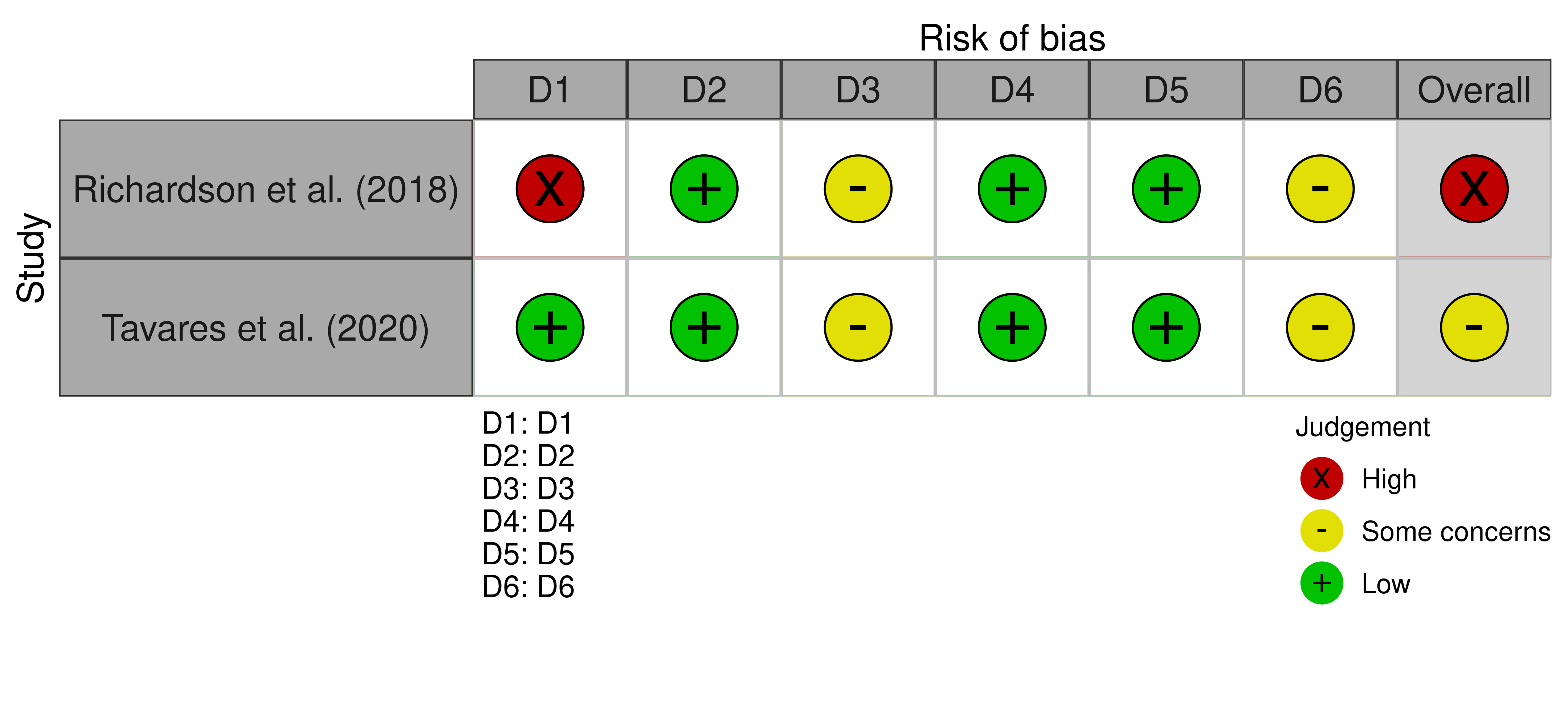

Supplement: S3 File — (TIF) [file pone.0294529.s004.tif]

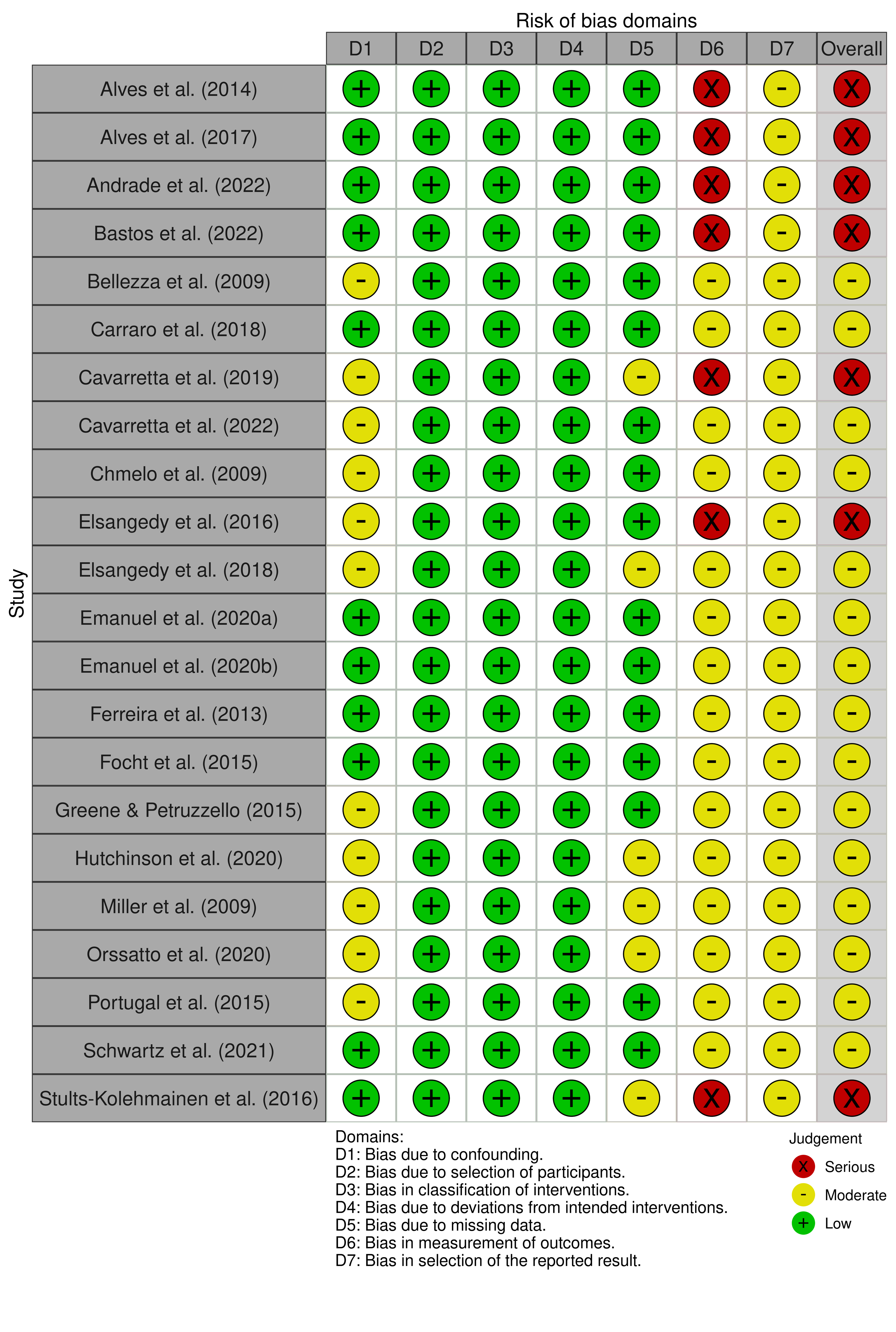

Supplement: S4 File — (TIF) [file pone.0294529.s005.tif]
