# Supplementary material for: Assessing affective valence and activation in resistance training with the feeling scale and the felt arousal scale: A systematic review
Source: PLoS One. 2023 Nov 16;18(11):e0294529. doi: 10.1371/journal.pone.0294529 (PMC10653427; doi:10.1371/journal.pone.0294529)
Supplement: Attachment — Submitted filename: PONE-D-23-08844_R1.pdf [file pone.0294529.s006.pdf]

The submitted manuscript aimed to assess how the feeling scale and felt arousal scale have been used in resistance exercise, using a systematic review approach. In this effort, a pre-registration in PROSPERO was made, and the PRISMA recommendations were followed. Additionally, bias assessment instruments (e.g., ROBINS) were used to evaluate the quality of the studies that met the inclusion criteria. Generally, I commend the authors for the adequate methodological approach developed.

The manuscript focused on understanding of two well know scales used in the affective response studies in a relevant context of practice. Although a very specific topic of interest, this review may help advance the much-needed topic of how to use and assess affective responses.

Generally, I found the manuscript to be well-written and organized. The results suggest that the FA and FAS are useful but depict several concerns given the research produced on the topic. Although I have no major concerns about this manuscript, I would like the authors to clarify the following:

Introduction

How would sex be considered an individual characteristic to affect the FS and FAS use in RT? Shouldn't these scales be interpreted the same way in both?

Method

Why an IMC > 34,9 was used? Does this contrast with the inclusion criteria of "apparently healthy individuals"'

Why effect size and power calculation were extracted from each study if no meta-analysis was performed?

There seems to be a typo in the ROBINS-I description (remove the dash in the extended form)

Discussion

"Although vastly different exercise intensities could be observed across the 27 studies included in this review, the FS and FAS successfully measured core affect". In fact, this statement clashed with the following arguments that state that methodological issues impeded the assessment of core affect. Perhaps here you should say "the affective response" rather than "core affect".

Apart from the FS and FAS, there are other instruments to assess the affective response. Should these be contemplated here (or in the limitations), as an alternative that can preset, perhaps, better support for its use?

Conclusion
Typo: the word "scales" in the first sentence is redundant. Also in this line, once again, perhaps saying "core affect" is not the accurate construct. Given that some

studies applied the FS and FAS with other timings that may not be adequate, what the authors are mentioning here is the affective response.
